# Pediatric Rotary Endodontic Files in Primary Teeth (2000–2025): A Scoping Review of Design, Evidence, and Clinical Use

**DOI:** 10.7759/cureus.85775

**Published:** 2025-06-11

**Authors:** Kavitha Swaminathan, Senthil Kumar Palanimuthu, Ganesh Rajendran, Monika Sri SS, Selvakumar Haridoss, Kritiga Kumar, Shalini B, Sushmita Shan

**Affiliations:** 1 Pediatric and Preventive Dentistry, Sri Ramachandra Institute of Higher Education and Research, Chennai, IND; 2 Pedodontics and Preventive Dentistry, Primary Health Care Corporation, Doha, QAT; 3 Pediatric and Preventive Dentistry, Al-Baha University, Al-Baha, SAU; 4 Pediatric and Preventive Dentistry, Sri Ramachandra Dental College and Hospital, Chennai, IND

**Keywords:** canal shaping, finite element analysis, kedo-s, niti rotary files, obturation quality, pediatric endodontics, primary teeth, pro af baby gold, rotary instrumentation, scoping review

## Abstract

Rotary instrumentation is increasingly popular in pediatric endodontics, but the evidence for pediatric-specific systems remains fragmented. This scoping review aimed to map the design features, clinical outcomes, and evidence gaps of rotary file systems used in primary teeth. We conducted a systematic search of PubMed, Scopus, Web of Science, and Google Scholar for studies published between 2000 and 2025. We included in vitro, clinical, and imaging-based studies, reviews, and case reports. Data on file design, study characteristics, and outcomes were extracted and synthesized. From 424 records, 111 studies were included. The results showed that pediatric-specific rotary systems (e.g., Kedo-S, Pro AF Baby Gold, Kedo-SG Blue) were consistently associated with reduced instrumentation time and improved canal shaping compared to manual techniques. Pediatric-specific rotary files demonstrate clear procedural advantages over manual methods. However, significant heterogeneity in study quality and limited global representation necessitate cautious interpretation. Future research should prioritize multicenter randomized controlled trials with long-term follow-up to provide higher-quality evidence.

## Introduction and background

The preservation of primary teeth is essential not only for functional and esthetic purposes but also for guiding the eruption of permanent successors and maintaining arch integrity [1]. Pulpectomy remains a widely accepted treatment modality for non-vital primary teeth, particularly in cases of extensive carious involvement or trauma [2]. Traditionally, root canal instrumentation in pediatric dentistry has relied on manual stainless steel files. While effective, these manual instruments have notable drawbacks. They are time-consuming, are technique-sensitive, and can lead to prolonged chairside time and discomfort, which is often poorly tolerated by young patients [3]. Nickel-titanium (NiTi) rotary files, made from a highly flexible metal alloy originally designed for permanent teeth, offer advantages such as improved flexibility, greater efficiency, and better canal centering. However, their initial use in pediatric endodontics was approached with caution due to concerns about anatomical mismatches, procedural safety, and the need for specialized clinician training [4]. Despite these concerns, rotary instrumentation is now increasingly utilized in pediatric dental practice. This trend underscores the need for a critical examination of the technology's evolution, its modifications, and its supporting evidence base.

In the last decade, systematic reviews and meta-analyses have assessed the effectiveness of rotary instrumentation in primary teeth, often comparing these systems to manual or reciprocating alternatives [5]. Several studies have reported that rotary systems reduce instrumentation time and may enhance obturation quality. For instance, reviews by Gala et al. and Faghihian et al. suggest that pediatric rotary files such as Kedo-S and Pro AF Baby Gold demonstrate superior shaping ability and reduced procedural duration compared to manual instrumentation [6,7]. However, these findings warrant cautious interpretation. Many of the existing reviews are based on small sample sizes, rely heavily on in vitro studies with limited clinical translatability, or include studies with heterogeneous methodologies that hinder robust comparisons [8,9]. Moreover, most reviews focus narrowly on select rotary systems and overlook newer designs such as Endogal Kids and Prime Pedo, which limits the comprehensiveness of their findings. There remains a critical gap in understanding how different pediatric rotary systems compare across parameters such as mechanical design, material composition, and clinical outcomes in real-world settings.

Although the literature increasingly supports the role of rotary instrumentation in pediatric endodontics, no existing synthesis has comprehensively mapped all rotary file systems developed specifically for pediatric use between 2000 and 2025. Prior reviews tend to be fragmented, either limited to individual brands or narrowly focused on a single outcome such as shaping efficiency or postoperative pain. Furthermore, many fail to capture the evolution in file design, including heat-treated NiTi metallurgy, pediatric-specific tapers, and shorter working lengths tailored to primary root morphology. The geographic concentration of available research particularly in countries such as India and Egypt also raises concerns regarding generalizability to diverse clinical populations. A broader, more inclusive review is therefore necessary to contextualize technological advancements, evidence trends, and clinical relevance.

This scoping review aims to bridge this gap by cataloging all rotary file systems developed for pediatric endodontics between 2000 and 2025. It further seeks to summarize their technical design features, including taper, length, metallurgy, and motion type. Additionally, it describes the reported outcomes across clinical, in vitro, and imaging-based studies while identifying areas of evidence scarcity and recommending priorities for future research.

## Review

Methodology

This scoping review was conducted in accordance with the Joanna Briggs Institute (JBI) framework and reported as per the PRISMA-ScR (Preferred Reporting Items for Systematic Reviews and Meta-Analyses Extension for Scoping Reviews) guidelines. A protocol was prospectively registered in the Open Science Framework (OSF) (https://doi.org/10.17605/OSF.IO/3VSMQ), outlining eligibility criteria, data charting methods, and synthesis plans.

Eligibility Criteria

We included primary studies (randomized controlled trials [RCTs], in vitro, ex vivo, observational, and finite element analysis (FEA)/imaging-based analyses), systematic reviews, and narrative reviews published between January 2000 and May 2025 that assessed pediatric rotary endodontic file systems in primary teeth. Studies were included regardless of language and setting. Case reports were included only if they introduced novel file systems not covered in other study designs. Studies exclusively on permanent teeth or adult instrumentation systems were excluded.

Search Strategy

A comprehensive search was conducted across PubMed, Scopus, Web of Science, Embase, Cochrane, and Google Scholar using controlled vocabulary (MeSH) and free-text terms: "pediatric rotary files", "primary teeth endodontics", "NiTi instrumentation", and "pulpectomy". The search was last updated on May 7, 2025. The first 100 hits from Google Scholar were screened for gray literature. Bibliographies of relevant reviews were hand-searched.

Selection and Data Charting

The study selection process was managed using Rayyan, a web-based software designed for systematic reviews. Following automated duplicate removal within the software, two reviewers independently screened the titles and abstracts of the remaining records. Full-text articles were then assessed for eligibility, with any disagreements resolved by consensus. Data were charted using a pre-piloted Excel form capturing study characteristics (author, year, country), file systems evaluated, study design, sample/model, outcomes measured, key findings, and conclusions. Outcome domains were classified as procedural (e.g., instrumentation time, file fracture), radiographic (e.g., obturation quality, canal shaping), microbiological (e.g., colony-forming units [CFUs]), and patient-reported (e.g., post-operative pain, behavior).

**Figure 1:**
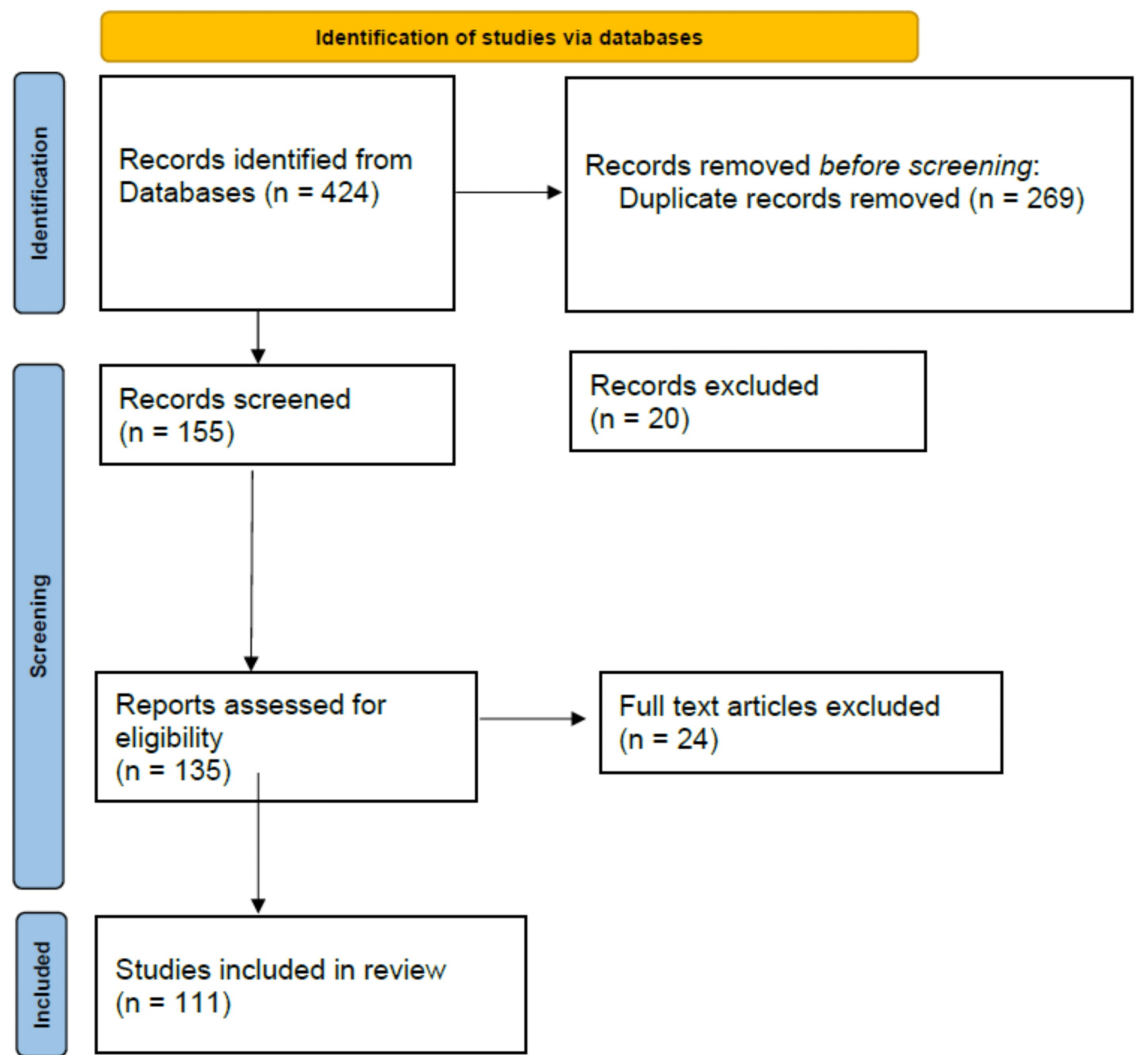
PRISMA 2020 flow diagram depicting the study selection process for the scoping review This PRISMA flow diagram illustrates the identification, screening, and inclusion process of studies for the scoping review on pediatric rotary file systems. A total of 424 records were identified through database searches. After removing 269 duplicates, 155 records were screened. Of these, 135 full-text articles were assessed for eligibility, with 24 excluded based on inclusion criteria. Ultimately, 111 studies were included in the final review.

Following database searches and removal of 269 duplicates, 155 titles/abstracts were screened, with 135 full texts assessed for eligibility. Ultimately, 111 articles were included in this scoping review. Articles from 2000 to 2025 were included to comprehensively evaluate the evolution and evidence base of pediatric rotary file systems (Figure 1).

Synthesis of Results

Results were synthesized descriptively and grouped by study design: in vitro studies, FEA-based studies, clinical studies (RCTs, observational), and review articles. Rotary systems were further categorized by generation, metallurgy, taper, and motion type. Summary tables were created to map available evidence, highlight commonly assessed outcomes, and identify underrepresented file systems.

Critical Appraisal

As per JBI and PRISMA-ScR guidance for scoping reviews, a formal risk of bias (e.g., RoB-2) or GRADE assessment was not performed, as the primary goal was to map the extent of the literature rather than to synthesize a quantitative effect estimate. However, the quality of the evidence was considered during the synthesis of results. Greater weight was given to evidence from studies with higher methodological rigor, such as RCTs and imaging-based analyses using Cone-beam computed tomography (CBCT) or nano-CT.

Results

**Figure 2:**
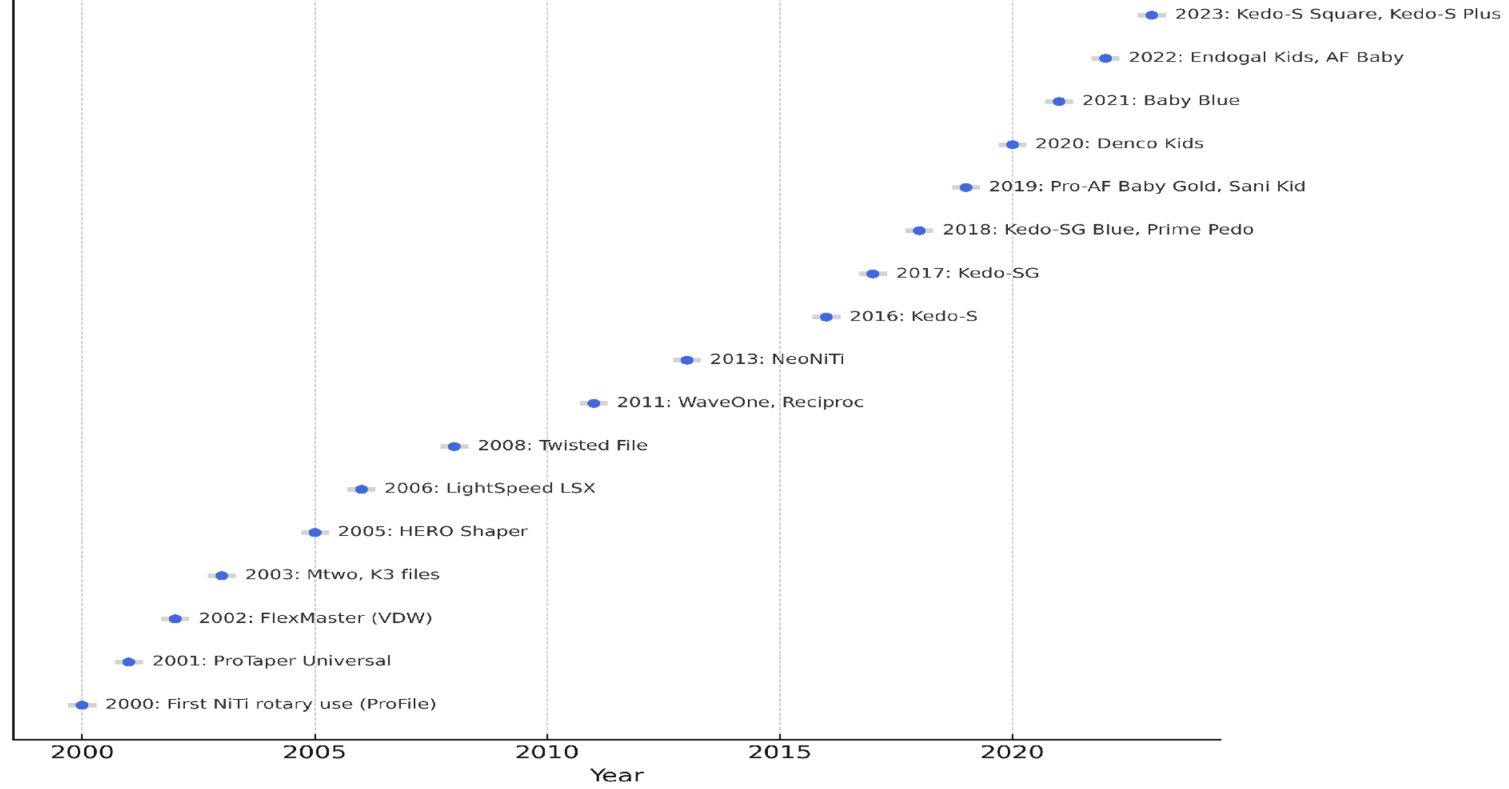
Evolution of rotary endodontic file systems from 2000 to 2025. The figure presents a chronological timeline of key rotary instruments, beginning with generalized systems originally designed for permanent teeth (e.g., ProFile, ProTaper Universal) and transitioning to pediatric-specific innovations such as Kedo-S (2016), Kedo-SG Blue (2018), and Kedo-S Plus (2023). This progression highlights the design evolution in metallurgy, taper, and working length tailored to primary teeth.

A total of 111 studies were included in this scoping review, published between 2000 and 2025. These included 26 RCTs, 62 in vitro studies, 2 FEA studies, 19 review articles, and 2 case reports. An updated timeline of pediatric rotary file system introductions (2000-2025) is presented in Figure 2.

While generalized NiTi systems such as ProFile and ProTaper were initially used in pediatric endodontics, the advent of pediatric-specific rotary systems beginning with Kedo-S in 2016 marked a significant shift. The technical specifications of these dedicated pediatric systems, including taper, metallurgy, and motion type, are summarized in Table 1. Disclosure of funding sources was largely absent across the included literature. Of the 111 studies included, only nine (8.1%) provided information on funding. The remaining articles did not report on the presence or absence of financial support for their research.

**Table 1:** Technical specifications of pediatric-specific rotary file systems introduced from 2016 to 2025. This table summarizes taper, motion type, metallurgy, and clinical considerations for commonly used pediatric rotary file systems. The information was primarily compiled from previously published reviews, namely Bonzanini et al. [9], Opi [10], and Kameswari et al. [11].

File System	Year Introduced	Taper (%)	Motion Type	Metallurgy	Clinical Use
Kedo-S	2016	4–6% (variable)	Rotary	Conventional NiTi	First pediatric-specific rotary system
Kedo-SG	2017	Variable	Rotary	NiTi (enhanced)	Improved design over Kedo-S
Kedo-SG Blue	2018	Variable (4–6%)	Rotary	Heat-treated NiTi (blue)	Greater flexibility for curved canals
Prime Pedo	2018	Variable	Rotary	NiTi	Designed for efficient canal shaping
Pro AF Baby Gold	2019	Variable	Rotary	Heat-treated NiTi	High fatigue resistance; good for narrow canals
Sani Kid	2019	Not reported	Rotary	NiTi	Pediatric file with limited published specs
Denco Kids	2020	Not reported	Rotary	NiTi	Pediatric adaptation by Denco
Baby Blue	2021	Variable	Rotary	Heat-treated NiTi	Pediatric file emphasizing stress resistance
Endogal Kids	2022	4%	Rotary	NiTi	Short length (16 mm); good for short roots
AF Baby	2022	4–6%	Rotary	Heat-treated NiTi	High flexibility; used in curved and narrow canals
Kedo-S Square	2023	Variable	Rotary	Enhanced NiTi Alloy	Increased fatigue resistance and centering
Kedo-S Plus	2023	Variable (4–8%)	Rotary	Heat-treated NiTi	Designed for advanced shaping; wider canal use [9-11]

In Vitro Studies

A total of 62 studies evaluated rotary instrumentation in primary teeth using in vitro or imaging techniques such as CBCT, scanning electron microscopy (SEM), nano-CT, and 3D printing [12-73]. These assessed outcomes such as instrumentation time, shaping ability, centering, debris extrusion, smear layer removal, and microbial reduction (Table 2).

**Table 2:** Summary of in vitro studies evaluating pediatric rotary endodontic files in primary teeth This table presents key study characteristics, including author, country, file systems evaluated, study design, sample details, assessed outcomes, and major findings. The table synthesizes in vitro evidence on rotary instrumentation in pediatric endodontics. 2D, two-dimensional; 3D, three-dimensional; BL, buccolingual; CAR, centering ability ratio; CBCT, cone-beam computed tomography; CFU, colony-forming units; EPD, electrophoretic deposition; GO, graphene oxide; LED, light-emitting diode; MD, mesiodistal; NiTi, nickel-titanium; PDL, periodontal ligament; RDT, remaining dentin thickness; SCT, spiral computed tomography; SEM, scanning electron microscopy; TBO, toluidine blue O

Author (Year)	Country	Study Design	File Systems Evaluated	Sample/Model	Outcome Measures Assessed	Key Findings	Conclusion/Clinical Implication	Remarks
Abushanan et al. (2025) [12]	Saudi Arabia	In vitro	Kedo-SG, Neoendo Pedoflex, Vortex Blue	120 simulated canals	Cyclic fatigue, SEM	Kedo-SG highest fatigue resistance	Kedo-SG comparable to Vortex Blue	Artificial models; lacks clinical realism
Chaudhary et al. (2025) [13]	India	In vitro	Kedo-SG Blue, Pro AF Baby Gold, hand K-files	30 central incisors	Debris extrusion	Rotary files extruded less debris	Rotary may reduce inflammation	Only anterior teeth studied
Bai et al. (2024) [14]	India	In vitro	Prime Pedo, DXL-Pro, Hand H/K	45 molars	Debris extrusion	Rotary extruded less debris	Rotary reduces complications	No microbial or long-term assessment
Eskibağlar and İpek (2024) [15]	Turkey	In vitro	Endoart Blue, M3 Blue, AF Baby, Hand	40 molars (distal roots only)	Apical debris extrusion	AF Baby extruded least debris; rotary < hand	Rotary safer for inflammation control	Single root type; small sample
Okasha et al. (2024) [16]	Egypt	In vitro	Manual K-files, Kedo-SG Blue, AF Baby Fanta	30 extracted primary canines (10 per group)	Cleaning efficacy (SEM), smear layer, debris scores, instrumentation time	Rotary groups (Kedo-SG and Fanta) showed significantly better cleaning and less smear layer than manual K-files. Kedo-SG was faster (1.65 min) than Fanta (1.85 min) and both were faster than K-files (3.09 min).	Rotary systems are more efficient in cleaning and save chair-side time in pediatric endo	Ex vivo resin canal simulation; SEM limited to surface evaluation; lacks bacterial/microbial comparison
Amin et al. (2024) [17]	Egypt	In vitro	Kedo-S, M Pro Pedo, ProTaper Gold	72 extracted primary molars	Cleaning, bacteria, obturation, time	M Pro fastest; ProTaper Gold best obturation	M Pro efficient but obturation suboptimal	Limited to palatal canals; no clinical behavior
Surme et al. (2024) [18]	Turkey	In vitro	X-Baby, miniScope, EndoArt Pedo Gold/Blue	80 simulated curved canals	Cyclic fatigue	EndoArt Pedo Blue highest fatigue resistance	Advanced NiTi files are durable	Simulated canals only; lacks hydration/movement factors
El-Desouky et al. (2024) [19]	Egypt	In vitro (CBCT)	Kedo-S, Kedo-SH, Kedo-SG	60 extracted molars	Canal taper, debris removal	Kedo-SH had best shaping and cleaning	File choice influences clinical success	Radiographic evaluation only; no clinical trial
Khalil and Samir (2024) [20]	Egypt	In vitro (CBCT)	K-files, Kedo-S Plus, Hyflex CM, Race Evo	140 extracted primary molars	Dentin thickness (middle third)	No significant difference among groups	All rotary systems equally effective	Distal roots only; no apical/coronal data
Kesri et al. (2024) [21]	India	In vitro	Pedoflex, Kedo-SH, Manual K-files	45 molars	Cutting efficiency via ink removal	Pedoflex > Kedo-SH > Manual	Pediatric rotary superior for cutting	Cleaning and obturation not studied. India ink lacks biological debris simulation
Panja et al. (2024) [22]	India	In vitro (SEM)	Kedo-SG rotary file (NiTi) coated with graphene oxide	10 NiTi pediatric rotary instruments	Surface topography via SEM, uniformity of GO coating	Graphene oxide coating via EPD showed uniform, continuous, multilayered GO sheets improving surface structure	GO-coated pediatric rotary files exhibit smoother surface morphology, potentially reducing fracture risk	Only surface morphology assessed; no mechanical or clinical testing performed; lacks coating thickness quantification
Suresh et al. (2024) [23]	India	In vitro (Nano-CT)	Kedo-S Plus, Kedo-SG Blue, hand K-files	60 extracted primary mandibular second molars (20/group)	Volumetric change in root canal space (nano-CT based)	Kedo-SG Blue showed the highest canal enlargement (8.85%), followed by Kedo-S Plus (6.14%) and hand K-files (1.24%). Rotary systems had significantly higher canal shaping ability than hand files.	Rotary systems offer superior volumetric preparation in primary teeth; Kedo-SG Blue achieved most uniform shaping	No clinical validation; limited to extracted teeth with wide canals; only volume changes measured—no microbial or obturation outcome considered
Vishwanathaiah (2024) [24]	Saudi Arabia	In vitro (CBCT)	Hand K-files, ProTaper, Kedo-SG Blue	30 extracted human primary second molars (10/group)	Canal volume (pre-/post-instrumentation), obturation volume (CBCT)	Kedo-SG Blue achieved the highest canal volume change post-instrumentation and post-obturation. ProTaper ranked second, followed by hand K-files. All intergroup differences were statistically significant (p = 0.047 to p = 0.001), with Kedo-SG Blue demonstrating the greatest reduction in post-instrumentation canal volume.	Kedo-SG Blue is most efficient in canal preparation and obturation in primary molars compared to ProTaper and hand files	No clinical validation; root curvature not standardized; only mesiobuccal root of second molars assessed; 3D obturation voids not evaluated
Eskibağlar and Özata (2024) [25]	Turkey	In vitro (Experimental)	ProTaper Ultimate (PTU), TruNatomy Prime (TRN)	With vs without glide path; with vs without resorption	80 extracted primary molars (n=10 per group)	Glide path usage significantly reduced apically extruded debris (p<0.001). Presence of resorption significantly increased debris extrusion (p<0.001). File system type (PTU vs TRN) not statistically significant.	Glide path preparation is effective in reducing apical debris extrusion in primary molars. Root resorption increases debris extrusion regardless of file system. TRN and PTU files performed similarly.	While the study effectively isolates the influence of glide path and resorption, its applicability to pediatric clinical practice may be limited by anatomical variability and lack of behavioral context
Abdelkafy et al. (2023) [26]	Egypt	In vitro (CBCT-based)	ProTaper Next (regular), AF Baby (Fanta), Kidzo Elephant (Poldent)	18 root canals (6 per group) from extracted primary molars	Canal transportation and centering ratio at cervical, middle, apical levels (BL and MD directions)	No significant difference in BL direction across groups. In MD direction at cervical level, ProTaper Next showed significantly higher canal transportation than pediatric rotary files. No significant difference in centering ratio in any group.	Pediatric rotary files (AF Baby and Kidzo Elephant) better preserve cervical canal anatomy mesiodistally than ProTaper Next	Small sample size; only CBCT imaging used; clinical correlation and long-term outcomes not studied
Fernandes et al. (2023) [27]	Brazil	In vitro	Manual K-files, Manual NiTi (ProDesign M), Rotary NiTi (ProDesign Logic)	60 artificial primary molars (stock teeth), 20 per group	Instrumentation time, quality of obturation, taper, flowability	Rotary files (ProDesign Logic) showed significantly shorter prep time (202.3s) than manual NiTi (307.0s) and manual K-files (383.5s); all methods equally effective in shaping and obturation quality	Rotary technique reduces operating time and improves standardization in pediatric root canal prep	Artificial teeth used; not generalizable to clinical anatomy; no microbial or post-operative outcomes evaluated
Öz et al. (2023) [28]	Turkey	Ex vivo	Rotary, reciprocating, hand files	Extracted molars	Bacterial reduction (*E. faecalis*)	All effective; rotary > hand	Rotary improves disinfection	Limited clinical simulation ex vivo only
Suresh et al. (2023) [29]	India, Saudi Arabia	In vitro	Hand K-files, Kedo-S Plus, Kedo-SG Blue	60 extracted primary maxillary central incisors (20 per group)	Apical debris extrusion (Myers and Montgomery model)	Kedo-S Plus: least extrusion (0.6561 mg), Kedo-SG Blue: moderate (0.1021 mg), hand K-file: highest (1.9963 mg)	Rotary files extrude significantly less debris than hand files; Kedo-S Plus performs best	In vitro only; lacks periapical tissue simulation; anterior straight canals only; needs in vivo validation
Jome et al. (2023) [30]	India	In vitro	Pedoflex, Pro AF Baby Gold, Kedo-SG Blue	15 extracted canines	Debris score, smoothness	Pedoflex had best results	Suitable for anterior canals	Very small sample; only anterior teeth used
Bal and Aksoy (2023) [31]	Turkey	In vitro (2D Simulation)	Endoart Pedo Smart Gold (EPSG), Endoart Smart Gold (ESG)	28 simulated curved resin canals	Resin removal (inner/outer), centering ratio, aberration presence	EPSG removed significantly less resin and had superior centering at coronal third; fewer aberrations than ESG	Pediatric-specific rotary file (EPSG) preserved canal anatomy better	Simulated model does not reflect true clinical tissue behavior; heat-softening of resin noted as limitation
Yehya et al. (2023) [32]	Egypt	In vitro	Manual K-files, Kedo-S Square, Kedo-S SG Blue	30 extracted primary maxillary second molars (60 canals)	Cleaning efficacy using India ink removal and clearing technique	Rotary groups (Kedo-S Square and Kedo-S SG Blue) had significantly better cleaning than manual files; no difference between the two rotary groups; apical cleaning least effective	Pediatric rotary files provide better canal cleaning than hand K-files; both systems performed similarly	No instrumentation time measured; apical cleaning not significantly different; India ink may not reflect clinical pulp tissue
Gucyetmez Topal et al. (2023) [33]	Turkey	In vitro (3D printed + CBCT)	VDW.ROTATE™, EdgeTaper Platinum™, Manual K-files	66 CBCT-based resin-printed second primary molars (22 per group)	Canal volume and area change, untouched canal surface area, instrumentation time	No significant difference in canal volume/area among groups. VDW.ROTATE™ had the lowest untouched canal surface area and shortest instrumentation time. EdgeTaper Platinum™ was faster than hand files but less efficient than VDW.ROTATE™.	VDW.ROTATE™ outperformed in shaping ability and time efficiency. 3D-printed resin teeth provide a reproducible model for standardized testing.	Printed teeth do not replicate dentin hardness/radiopacity; anatomical variations absent; clinical applicability requires caution
Özdoğru and Keskin (2023) [34]	Turkey	In vitro (resin)	Kiddy, AF Baby, One G + AF Baby, hand K-files	50 resin block canals	Canal transportation, time	Kiddy best centered; glide path improved AF Baby performance	Glide path improves shaping efficiency	Resin blocks lack tooth resistance and irrigation simulation
Shanker and Patil (2023) [35]	India	In vitro (microbiological + radiographic)	Kedo-SH (hand NiTi), Kedo-SG Blue (rotary), Pro AF Baby Gold (rotary)	51 extracted primary molars divided into 3 groups of 17 teeth	Microbial reduction (*E. faecalis *CFUs), obturation quality (T-scoring: taper, density, length)	Pro AF Baby Gold showed greatest microbial reduction and highest ideal obturation (length, density, taper). Kedo-SG Blue also effective, better than Kedo-SH. T-scores: group III > II > I. All groups showed statistically significant CFU reduction post-instrumentation.	Rotary files outperform hand files in shaping and cleaning; Pro AF Baby Gold delivers optimal obturation and disinfection	Radiographs were 2D; no CBCT validation; simulated conditions; Metapex was the obturation material in all samples.
Faus-Llácer et al. (2022) [36]	Spain	In vitro (Micro-CT)	Endogal Kids vs Reciproc Blue	60 canals in molars	Dentin removal (3 levels)	Reciproc preserved more coronal dentin	Reciproc less invasive	Only dentin evaluated; no obturation or cleaning
Tofangchiha et al. (2022) [37]	Iran	In vitro	Kedo-S, RaCe, hand K-files	120 root canals of primary second molars	Cleaning efficacy in apical, middle, and coronal thirds using India ink method	Kedo-S showed significantly superior cleaning in the coronal third compared to RaCe and hand files; no significant differences in middle/apical thirds	Kedo-S files enhance cleaning efficiency in coronal areas; advantageous in pediatric pulpectomy	In vitro only; India ink is limited in quantifying biofilm or tissue debris; results may not generalize to clinical settings
Mohamed et al. (2022) [38]	Egypt	In vitro (CBCT)	Kedo-S Square	60 anterior teeth	Canal shaping, preparation accuracy	Good taper and shaping with rotary file	Rotary viable in anterior teeth	Not validated in vivo
Subramaniam et al. (2022) [39]	India	In vitro (SEM)	Kedo-S, Pro AF Baby Gold, K-files	60 anterior extracted teeth	Smear layer removal scores	Rotary removed more smear layer	Superior canal wall cleanliness with rotary	Only smear layer assessed; limited to anterior teeth
Peedikayil et al. (2022) [40]	India	In vitro	Kedo-SG Blue, Pro AF Baby Gold, Kedo-SH, ProTaper Hand	60 extracted single-rooted primary teeth (15 per group)	Amount of apical debris extrusion using Myers and Montgomery model	Kedo-SG Blue extruded the least debris (0.00187 μg) followed by Pro AF Baby Gold (0.00340 μg); hand files (Kedo-SH: 0.00413 μg; ProTaper: 0.00500 μg)	Rotary systems, especially Kedo-SG Blue, significantly reduce apical debris extrusion in primary teeth	Does not simulate in vivo anatomy or irrigation dynamics; results may vary with different canal morphologies and irrigants
Mahmoud et al. (2022) [41]	Egypt	In vitro (SEM)	Kedo-SG Blue, Wave One Gold, hand K-files	75 mandibular primary second molars, 25 per group	Dentinal crack formation in coronal, middle, and apical thirds	Cracks observed in 41.3% (WOG), 24.0% (Kedo-SG Blue), and 13.3% (hand K-file). - Hand files had significantly fewer cracks than rotary systems, especially in coronal and apical thirds.	Kedo-SG Blue produced fewer dentinal cracks than WOG. Manual files caused the least. Rotary systems still show promise but may increase crack risk.	No PDL simulation; SEM offers surface-level insights; clinical loading and cyclic fatigue not accounted for
Rosas et al. (2021) [42]	Brazil	In vitro	Manual H-file (Angelus), Sequence Baby Rotary, rotary + PDT	48 root canals of primary molars, 12 per group	Bacterial count (CFU/mL) of *E. faecalis *pre- and post-intervention using ANOVA and Dunnett’s test	All intervention groups significantly reduced *E. faecalis*. PDT group (G3) had the highest bacterial reduction (log reduction: 1.96), followed by rotary (1.58) and manual (1.36)	PDT + rotary instrumentation most effective in disinfection; supports PDT as adjunctive tool	No antimicrobial irrigants used (only saline); ex vivo model; laser PDT lacks clinical standardization for primary teeth
Singh et al. (2022) [43]	India	In vitro (CBCT)	NiTi K-files, ProTaper Next (PTN), OneShape (OS), WaveOne (WO)	60 canals from 44 extracted primary teeth (15 per group)	Canal transportation, CAR, RDT, dentinal cracks, instrumentation time (min)	PTN had least canal transportation; OS best centering ability. NiTi K-files preserved maximum dentin and caused minimum cracks. WO had shortest instrumentation time (1.83 min).	Rotary files such as PTN and OS maintain canal shape better than hand files; WO offers speed advantage; K-files preserve more dentin	CBCT provided objective evaluation; study lacked evaluation of debris removal or obturation quality; only short canals studied
Swaminathan et al. (2022) [44]	India	In vitro (CBCT)	Kedo-S vs Mtwo rotary files	50 extracted mandibular primary first molars (25 per group)	Instrumentation time, dentin removal (mesial/distal), lateral perforation (CBCT)	Kedo-S showed significantly shorter instrumentation time (53.4 s) than Mtwo (192.28 s). No significant difference in dentin removal. Mtwo caused more apical perforations (28%) than Kedo-S (12%).	Kedo-S is safer and faster, with lower perforation risk in primary teeth than traditional rotary systems	Focused only on distal canals; no obturation or cleaning efficacy evaluated; needs validation in curved canals or clinical setting
Haridoss et al. (2022) [45]	India	In vitro (CBCT)	Kedo-S (pediatric rotary), Mtwo (NiTi)	50 extracted primary mandibular first molars (25 per group)	Canal transportation and centering ability (at 2, 4, 6 mm from CEJ)	Both file systems showed minimal and comparable transportation and maintained centering ability. Kedo-S showed slightly more distal transport. Mtwo showed better centering at apical level, but not significant statistically.	Kedo-S and Mtwo are both safe for primary root canal shaping with minimal iatrogenic risk	In vitro CBCT-based data only; lacks long-term follow-up; clinical correlation needed with real pulp tissue or curved canals
Islam et al. (2021) [46]	Cyprus	In vitro (CBCT)	ProTaper Gold, RaceEvo, R-Motion	60 curved primary molars	Canal transportation, dentin removal	ProTaper removed more dentin; RaceEvo preserved anatomy	Newer rotary systems better for canal preservation	Not clinically validated
Güçyetmez Topal et al. (2021) [47]	Turkey	In vitro (CBCT)	EndoArt Ni-Ti Gold Pedo Kit vs hand K-files	30 extracted primary molars (15/group) with 7mm root length	Canal transportation (BL and MD), instrumentation time, dentin removal (coronal, middle, apical thirds)	No significant difference in canal transportation or instrumentation time. EndoArt removed significantly more dentin at coronal and middle thirds (p<0.05). Both systems kept transportation < 0.15 mm (clinically acceptable).	EndoArt rotary system provides more conical canal shaping and efficient dentin removal in coronal/middle thirds	Small sample; no microbial/debris evaluation; no long-term obturation or clinical performance tracked
Rathi et al. (2021) [48]	India	In vitro	Kedo-S, Pro AF Baby Gold	20 extracted molars	Cleaning, apical extrusion	Pro AF had better cleaning, less debris extrusion	Consider Pro AF over Kedo-S	No clinical correlation or follow-up
Pawar et al. (2021) [49]	India	Ex vivo Study	Hand K-files, Kedo-S, XP-endo Shaper	45 extracted primary canines (n=15/group)	Apical debris extrusion, instrumentation time	XP-endo Shaper extruded least debris (0.84 mg), followed by Kedo-S (1.20 mg) and hand K-files (2.13 mg). XP-endo also had the shortest instrumentation time (2.38 min).	Motorized files reduce debris extrusion and save time; especially useful in pediatric endodontics	Only anterior straight-rooted teeth used; findings not generalizable to molars or resorbed teeth
Waly et al. (2021) [50]	Egypt/Saudi Arabia	Ex vivo (CBCT)	Kedo-S, Pro AF Baby Gold, hand K-files	72 canals from extracted 2° molars	Transportation, dentin thickness, centering ratio	Rotary systems faster; shaping comparable	Efficient with no compromise in canal shape	CBCT-based; lacks biological data
Alfadhli et al. (2021) [51]	Saudi Arabia, Egypt	In vitro	K-files (manual), Kedo-SG (rotary)	58 canals from primary molars (31 rotary, 27 manual)	Cleaning efficacy (India ink), instrumentation time	Kedo-SG group had significantly shorter preparation time (58 s vs 91 s) and higher complete canal cleaning (Score 0: 12 vs 2)	Rotary files improve cleaning efficacy and reduce time in pulpectomy	Resin clearing method; limited to ex vivo conditions; India ink is not equivalent to real pulp tissue
Kalita et al. (2021) [52]	India	In vitro	Kedo-S, ProTaper, hand K-files	120 root canals	Cleaning efficacy, time	Kedo-S best in coronal/middle thirds; fastest file	Improves cleaning and saves time	Apical cleaning not significantly better
Eldemery et al. (2021) [53]	Egypt	In vitro (CBCT)	Manual SS K-files vs AF™ Baby Rotary	40 roots from mandibular primary molars (20/group)	RDT at coronal, middle, apical thirds using CBCT	Rotary AF™ Baby File removed significantly less dentin than manual files at all levels; Manual group had almost double % of dentin loss (35–37% vs 17%) at all thirds.	Rotary files better preserve radicular dentin structure and are more suitable for curved primary roots	Only dentin thickness measured; no evaluation of canal centering, cleaning efficacy, or post-operative behavior
Preethy et al. (2019) [54]	India	In vitro	Hand K-files, K3, Kedo-S	36 primary canines	Apical debris extrusion	Kedo-S had significantly less debris extrusion	Reduces risk of extrusion-associated pain	Only anterior teeth assessed
Katge et al. (2019) [55]	India	In vitro	Prime Pedo, DXL-Pro, H-files	60 root canals in primary molars	Cleaning efficacy using India ink	Prime Pedo and DXL-Pro > H-files (coronal, apical thirds)	Pediatric rotary files enhance cleanliness	No difference in middle third; ink lacks clinical correlation
Akkam et al. (2019) [56]	Saudi Arabia	In vitro (CBCT)	K-files, Rotary NiTi, Kedo-S	30 extracted molars	Canal enlargement, obturation	Rotary systems > manual for shaping and fill	Enhanced rotary shaping outcomes	Poster; peer-review status unclear
Shaikh and Goswami (2018) [57]	India	In vitro (CBCT)	Revo-S, Sonic MM1500, hand K-files	75 molars	Centering, canal shaping	Sonic better at apical shaping; rotary better centering	Use combined approach	Time and clinical outcomes not measured
Radhika et al. (2017) [58]	India	In vitro (CBCT)	Hand NiTi vs rotary (unspecified)	40 primary molars	Canal shaping, centering	Rotary showed better centering and shaping	Rotary safer in primary canal prep	File brands not mentioned; outdated scope
Katge et al. (2016) [59]	India	In vitro	H-files, Mtwo	90 canals	Cleaning, instrumentation time	Similar cleaning; Mtwo slower	Manual still relevant in some cases	No obturation or post-operative assessment
Selvakumar et al. (2016) [60]	India	In vitro (CT)	Stainless steel K, K3 (0.02/0.04)	75 molars	Dentin removal, time, perforation risk	K3 (0.02) preserved more dentin	Prefer low taper files in primary molars	Older files; newer pediatric systems not compared
Katge et al. (2014) [61]	India	In vitro	K-files, ProTaper, WaveOne	120 root canals from 84 primary molars	Cleaning efficacy and time	WaveOne best in coronal/middle thirds	Reciprocating better than manual	Older NiTi tech; pediatric-specific files not tested
Selvakumar et al. (2014) [62]	India	In vitro (SCT)	K-files, K3 (0.02%, 0.04%)	75 primary molars	Canal transportation, centering	K3 (0.02%) had best centering and least transportation	Low-taper K3 safe for primary molars	Old tech; no newer pediatric rotary systems used
Prabhakar et al. (2014) [63]	India	In vitro experimental	Twisted Files (TF), ProTaper	30 extracted primary molars (15 per group)	Cutting efficiency (Indian ink removal scored 0–3); statistical comparison via Mann-Whitney U test	Twisted files achieved complete ink removal in 53.3% vs 13.3% with ProTaper (p=0.02); more uniform cleaning with TF; ProTaper removed more dentin unevenly	TF files showed superior cutting efficiency in primary molars and are suitable for pediatric endodontics	Small sample; subjective scoring method (ink); no evaluation of canal transportation or time; adult files used; lack of long-term performance or fracture resistance data
Pinheiro et al. (2014) [64]	Brazil	In vitro microbiological study	Manual K-files vs ProTaper rotary (with PDT combinations: TBO/laser, fuchsin/LED, fuchsin/halogen light)	20 extracted primary molars (*E. faecalis* infected, 10 per group)	CFU count of *E. faecalis *before/after instrumentation and PDT (Wilcoxon, t-test, Kruskal-Wallis)	Both manual and rotary instrumentation significantly reduced *E. faecalis *counts. PDT (all 3 combinations) further enhanced bacterial reduction. No significant difference between rotary and manual groups in baseline or post-PDT counts.	PDT is effective as an adjunct to both manual and rotary instrumentation in reducing *E. faecalis *in primary teeth	Small sample size (n=10 per group); adult rotary system used; focused only on one bacterial species (*E. faecalis*); no shaping or obturation outcome assessed; PDT not yet common in clinical pediatric endo
Musale and Mujawar (2013) [65]	India	In vitro experimental	ProTaper, ProFile, Hero Shaper (all rotary) vs hand K-files	60 extracted primary mandibular second molars (15 per group)	Shaping ability (CBCT taper assessment); cleaning efficacy (India ink removal); instrumentation time; instrument distortion	Rotary files produced significantly better canal taper than hand files (p<0.05). Cleaning efficacy: ProTaper > Hero Shaper > ProFile > K-file. K-files had highest mean time (20.7 min) vs rotary (5.6–8.9 min). No distortion in rotary groups; 1 distorted K-file.	Rotary instrumentation yielded faster preparation, cleaner canals, and better taper; beneficial for pediatric clinical use	Adult rotary systems used; operator was a novice with rotary use (bias potential); only one distortion event may not reflect real-world fatigue; CBCT offers high fidelity but limited clinical replication of working conditions
Azar et al. (2012) [66]	Iran	In vitro experimental	Mtwo, ProTaper, Manual K-files	80 extracted mandibular primary molars (160 canals)	Cleaning efficacy via India ink scoring (0–3) in coronal, middle, and apical thirds	No significant difference overall; ProTaper better in coronal/middle thirds, Mtwo more uniform; none cleaned apical third effectively	Adult rotary files showed comparable cleaning to hand files; adaptation of rotary systems in pediatric endo is feasible	Small sample; adult systems tested in primary teeth; subjective scoring system; apical cleaning poor; results may not generalize to newer pediatric systems
Pinheiro et al. (2012) [67]	Brazil	In vitro experimental (ex vivo microbiological + SEM)	ProTaper (rotary), K-files (manual), Hybrid (manual + rotary)	15 primary molars infected with *E. faecalis*, embedded and instrumented	Instrumentation time; microbial reduction (*E. faecalis*); SEM analysis of debris and smear layer	Hybrid technique showed highest bacterial reduction (99.58%), but took longest time. Rotary files had shorter time and less smear layer. Manual had more smear layer but less debris.	Rotary NiTi instrumentation is faster and more efficient than manual techniques; hybrid improves disinfection but is time-consuming	Small sample (n=5 per group); no long-term clinical validation; rotary and hybrid both used ProTaper (adult system); unclear if clinical operator variability is reflected
Madan et al. (2011) [68]	India	In vitro experimental	ProFiles 0.04 taper (Dentsply) vs manual K-files (Kendo)	75 extracted primary molar root canals (with 2/3 root intact)	Cleaning efficiency via India ink clearing; Instrumentation time (chronometer)	ProFiles cleaned coronal third better; K-files better in apical third (p<0.001). No significant difference in middle third cleaning. K-files faster in both arches (p<0.001).	Rotary ProFiles offer effective coronal shaping, but K-files excel in apical cleaning. Manual files also saved time.	India ink method lacks microbial relevance; instrumentation time may reflect operator bias; adult rotary system tested in primary roots without taper adaptation for narrow canals
Azar and Mokhtare (2011) [69]	Iran	In vitro Experimental (Ex vivo)	Mtwo rotary system vs manual K-files	70 primary and 70 permanent molars; 120 canals ink-stained and analyzed	Cleaning ability (India ink score in 3 canal thirds); instrumentation time; file fracture incidence	No significant difference in cleaning ability in any third (p > 0.05); rotary files significantly faster (primary: 259s vs 434.7s; permanent: 414.6s vs 831.6s); minimal file fracture	Mtwo files are safe and effective for primary molars; faster than hand files and comparable in cleaning	Adult files (Mtwo) used in pediatric canals; India ink method may not correlate with microbial debridement; no evaluation of canal transportation; no clinical correlation provided
Nazari Moghaddam et al. (2009) [70]	Spain	In vitro	ProFile vs hand K-files	60 single-rooted primary teeth	Time, shaping	Rotary faster, more tapered shape	Better canal preparation	Outdated tech (ProFile); no clinical validation
Crespo et al. (2008) [71]	Spain	In vitro	ProFile vs hand K-files	60 single-rooted primary teeth	Time, shaping	Rotary faster, more tapered shape	Better canal preparation	Outdated tech (ProFile); no clinical validation
Kummer et al. (2008) [72]	Brazil	Ex vivo in vitro study	Hero 642 (NiTi rotary) vs hand K-files	80 extracted human primary teeth (40 per group), embedded in endodontic cubes	Dentin removal (mm² via digital image analysis); instrumentation time (min); canal shape; root perforation incidence	Manual files removed more dentin at all levels (p < 0.05). Rotary files significantly faster (all tooth groups, p < 0.05). More root perforations in areas of advanced resorption (especially mid-root and lingual roots). Rotary resulted in more regular shaping.	Rotary instrumentation is faster and conserves dentin better than manual techniques. Care needed near resorbed areas due to risk of perforation	Complex methodology using embedded cube models, which while accurate, is not replicable in clinical settings. Hero 642 is an adult system. Variable canal anatomy and extent of resorption not fully standardized or accounted for.
Nagaratna et al. (2006) [73]	India	In vitro experimental	ProFile 0.04 taper (Dentsply), Stainless Steel K-files	20 primary mandibular second molars (group I) and 20 permanent molars (Group II)	Preparation time; instrument failure (deformation or fracture); canal shaping: flow, taper, and wall smoothness via elastomeric impressions	Rotary files significantly reduced instrumentation time (primary: 8.51 min vs 13.39 min; permanent: 9.91 min vs 15.99 min, p<0.001). Better canal shaping (flow, taper, and smoothness) in rotary group for most canals. K-files showed more deformation; rotary files had more fracture events.	NiTi rotary (ProFile) showed superior performance in shaping and speed for both primary and permanent molars, though file fractures were noted	Adult rotary files tested in primary teeth; small sample size (n=10 per subgroup); artificial model may not replicate clinical variability; use of elastomeric impression for internal canal morphology is uncommon in current literature

Pediatric-specific rotary systems such as Kedo-S, Kedo-SG Blue, Pro AF Baby Gold, Prime Pedo, and PedoFlex consistently outperformed manual files in time efficiency, shaping accuracy, and bacterial load reduction was studied by Kalita et al. [52], Amin and Wassel [17], Okasha et al. [16], and Gucyetmez Topal et al. [33]. Advanced evaluations using CBCT and nano-CT confirmed superior canal volume changes and centering with rotary files [23,24,33]. Smear layer removal was significantly better in SEM-based comparisons [39,41].

Rotary instrumentation also minimized apical debris extrusion ([13,40. Files such as Kedo-S Plus, VDW.ROTATE™, EndoArt Pedo Smart Gold, and Graphene-coated prototypes showed advancements in fatigue resistance and cutting efficiency [22,23,31,33]. The 62 in vitro studies demonstrate that pediatric rotary files consistently achieve better canal shaping, shorter instrumentation time, and reduced procedural risks compared to manual systems.

FEA-Based Studies

Only two studies employed FEA modeling to simulate stress and fatigue performance in pediatric rotary files. Monika Sri et al. found that Pro AF Baby exhibited the lowest stress levels and highest fatigue resistance across simulated canal curvatures (30°, 60°, 90°) [74]. Manivannan et al. reported that Kedo-SG showed highest flexibility, while Pro AF Baby withstood bending stress without yield [75] (Table 3).

**Table 3:** Summary of FEA studies on pediatric rotary file systems This table presents two FEA-based studies evaluating stress distribution, fatigue resistance, and flexibility of pediatric rotary instruments in simulated primary molar canals. It includes study characteristics, 3D model details, mechanical outcomes, and implications for pediatric endodontics. 3D, three-dimensional; CAD, computer-aided design; FEA, finite element analysis; MPa, megapascal Von Mises stress: a calculated stress used to predict yielding of materials under complex loading.

Author (Year)	Country	Study Design	File Systems Evaluated	Sample/Model	Outcome Measures Assessed	Key Findings	Conclusion / Clinical Implication	Remarks
Monika Sri et al. (2025) [74]	India	FEA	Kedo-SG Blue-D1, Neoendo Pedo Flex, Pro AF Baby B2	3D models of primary molar canals with 30°, 60°, 90° curvature	Maximum stress (MPa), number of fatigue cycles to failure	Pro AF Baby showed lowest stress values and highest fatigue resistance across all canal curvatures. Stress increased with curvature: Kedo-SG showed highest stress at 90° (4063 MPa) and Pro AF the least (2022 MPa). Pro AF completed more cycles before fatigue failure.	Pro AF Baby rotary file is best suited for complex curvatures in primary molars due to superior fatigue resistance and lower stress concentrations	FEA simulations only; no clinical validation; anatomical variations and in vivo loading forces not fully replicated
Manivannan et al. (2024) [75]	India	In vitro (FEA)	Pro AF Baby, Kedo-SG, Neoendo Pedoflex	CAD-based FEA simulation using pediatric rotary files	Von Mises stress (bending/torsion), yield limit, flexibility	Kedo-SG showed highest flexibility; Pro AF Baby withstood bending without yielding	Pro AF Baby is ideal for narrow canals; Kedo-SG for curved canals	FEA lacks in vivo validation; depends on mesh and boundary condition accuracy

Despite the limited number, these FEA-based studies support the mechanical superiority of pediatric rotary systems under stress. However, findings must be interpreted cautiously due to the limitations of simulation fidelity.

Clinical Studies (RCTs and In Vivo Trials)

A total of 26 clinical studies (RCTs and in vivo trials) examined rotary instrumentation outcomes in children [76-101]. Studies originated mainly from India, Egypt, Iran, and other regions (Table 4).

**Table 4:** Summary of RCTs and clinical studies evaluating pediatric rotary endodontic file systems This table presents RCTs and prospective clinical studies comparing pediatric rotary and manual file systems in primary teeth. The studies assess clinical outcomes such as instrumentation time, obturation quality, postoperative pain, microbial reduction, and behavior outcomes across diverse clinical settings and populations. CBCT, cone-beam computed tomography; CFU, colony-forming units; DOM, dental operating microscope; NiTi, nickel-titanium; PSP, phosphor storage plate; RCT, randomized controlled trial; VCAS, Venham Clinical Anxiety Scale; WL, working length

Author (Year)	Country	Study Design	File Systems Evaluated	Sample/Model	Outcome Measures Assessed	Key Findings	Conclusion/Clinical Implication	Remarks
Al-Wesabi et al. (2025) [76]	Yemen	Clinical trial	Kedo-SG Blue, Kedo-S Square, H-files	72 children	Behavior, pain, instrumentation time	Rotary files improved behavior and reduced pain	Kedo-S Square recommended for efficiency	Short-term behavior only; no follow-up
Bohidar et al. (2024) [77]	India	RCT	Manual K-files, Kedo-S	36 primary molars	Pain over 72 hrs	Rotary group had significantly lower pain	Improves post-operative experience in kids	Subjective pain; no radiographic or microbial success
Jeepalyam et al. (2024) [78]	India	RCT	Kedo-SG Blue, Prime Pedo	50 primary molars	Instrumentation/obturation time, obturation quality	Kedo-SG Blue was faster and better in obturation	More efficient for pediatric pulpectomy	Single-center; no microbial or long-term outcome
Thakur et al. (2024) [79]	India	In vivo (RCT)	Prime Pedo™ rotary, DXL-Pro™ rotary, Manual H-files	51 primary mandibular molars in children aged 4–10 (17 per group)	Instrumentation time, quality of obturation (Coll and Sadrian criteria)	Rotary groups (Prime Pedo™: 65.05 ± 4.02 s, DXL-Pro™: 66.29 ± 4.45 s) had significantly shorter instrumentation times than manual (140.05 ± 5.54s). No significant difference in obturation quality between groups.	Pediatric rotary systems are efficient and reduce chair-side time; obturation quality remains comparable to manual files	Single-blinded study; short follow-up; 2D radiographic evaluation; lacks microbial or pain-related outcome tracking
Saxena et al. (2023) [80]	India	RCT	K-files vs Pro AF Baby Gold	60 primary molars	Post-operative pain, time, obturation	Rotary faster; no difference in pain or obturation	Rotary files help reduce chair time	Pain scoring subjective; no long-term evaluation
Kumar and Jeevanandan (2023) [81]	India	RCT	Hand K-files, Kedo-S Square, Kedo-S Plus	45 children (4–8 yrs); 15/group; single visit pulpectomy	Instrumentation time, obturation quality (Coll and Sadrian), post-operative pain (Wong-Baker), follow-up at 3, 6, 12 months	Kedo-S Plus had shortest time (57.5s), best obturation (100%), and lowest post-operative pain scores	Kedo-S Plus is superior for pediatric molars in clinical efficiency and comfort	Small sample; used PSP radiographs, which may not detect micro voids; lacks microbial or CBCT analysis
Kumar and Jeevanandan (2023) [82]	India	Prospective clinical study	Kedo-S Plus pediatric rotary file	100 primary molars (335 root canals) in 4- to 9-year-old children	Fracture incidence of rotary files (visualized under 8x magnification), canal location	Only 1 file fractured (1%) in 335 canals, specifically in the apical third of the mesiobuccal canal of a maxillary second molar in group D (used 12 times); no fractures in other groups	Kedo-S Plus rotary file has low fracture incidence even when reused multiple times; most fractures occur in curved, narrow canals	Single file design; findings limited to clinical visualization; no fatigue analysis or fracture propagation microscopy included
Arora Sowmya et al. (2023) [83]	India	RCT	Rotary vs Manual	40 mandibular molars	Clinical success, time	Rotary faster, both clinically effective	Rotary is efficient in practice	No behavioral scoring or obturation quality reported
Arora et al. (2023([84]	India	In vivo (RCT)	Hand K-files vs Pro AF Baby Gold	150 primary mandibular molars in 140 children (age 4–7)	Instrumentation time, obturation quality (Coll and Sadrian), post-operative pain at multiple intervals	Rotary instrumentation (Pro AF Baby Gold) required significantly less time (27.44 min vs 50.88 min), higher optimal obturation (76% vs 52%), less pain at 6 and 12 hrs	Rotary technique offers superior efficiency, less post-operative pain, better obturation in pulpectomy	Follow-up limited to 3 weeks; behavior and microbial clearance not evaluated; examiners used PSP not CBCT
Abdel Rahman et al. (2023) [85]	Egypt	RCT	Manual H-files, Kedo-SG Blue, Kedo-S Square	93 primary molars	Time, anxiety (VCAS scale)	Kedo-S Square had lowest anxiety and fastest time	Enhances cooperation in children	Only short-term outcome; no clinical performance tested
Patel et al. (2023) [86]	India	RCT	Hand K-files, HeroShaper, Kedo-S Square	60 mandibular molars	Time, obturation, pain perception	Kedo-S Square fastest; all had similar obturation and pain	Efficient option for short chair-time	Pain data subjective; no follow-up
Kumar and Rehman (2023) [87]	India	RCT: in vivo clinical study	Neo Endo Flex rotary files (group II), standard manual files (group I)	208 deciduous molars in children aged 3–8 years, split into two groups (98 and 90 treated teeth, respectively)	Clinical and radiographic success at 6 and 12 months	No significant difference between microscope and conventional groups in clinical (96.6% vs 97.7%) and radiographic (95.5% vs 98.8%) success at 12 months. DOM (microscope) group showed slightly better outcomes with fewer radiolucencies and resorption cases.	Dental operating microscope can enhance visualization and possibly outcomes in pediatric endo, but results are comparable with conventional treatment when protocols are standardized.	Microscope use adds cost and requires training; patient anxiety due to microscope setup not studied; long-term follow-up beyond 1 year not available
Thakur et al. (2023) [88]	India, Indonesia, UAE	RCT: in vivo clinical study	XP-endo Shaper (adaptive), Kedo-SG Blue (rotary), manual K-files	75 children aged 4–9 years (25 per group); mandibular primary molars	Post-operative pain at 6, 12, 24, 48, 72 hours (Wong-Baker scale)	- XP-endo group showed least post-operative pain at 6 and 12 hrs - Kedo-SG moderate pain - Manual files had highest pain at early time points - By 24 hrs, all groups showed minimal or no pain	XP-endo Shaper adaptive files significantly reduce early post-operative pain in primary molars; optimal for pediatric endo	Sample size limited; only short-term post-operative pain assessed; quality of obturation assessed only via 2D radiograph; no microbial analysis
Hadwa et al. (2023) [89]	Egypt	RCT (Triple-blinded)	Kedo-S-Square, Fanta AF™ Baby	Manual K-file	60 children, 3 groups (n=20 each)	- Instrumentation time (Kedo: 74.75s; Fanta: 76.6s; Manual: 106.2s) - CBCT-based obturation quality (Optimal fill: Kedo 85%, Fanta 75%, K-file 70%) - Post-operative pain scores at 6h, 12h, 24h, 48h lower in rotary groups (significant)	Rotary files (Kedo-S-Square and Fanta AF™ Baby) showed superior instrumentation efficiency, obturation quality, and less postoperative pain compared to manual K-files. Use of CBCT offered reliable 3D assessment.	While the study supports superior performance of rotary systems in terms of pain and efficiency, the small sample size and limited methodological detail (e.g., CBCT calibration, file reuse protocol) warrant cautious interpretation.
Babu et al. (2022) [90]	India	Randomized Clinical Trial (RCT)	Pedo-Flex, Kedo-S, Manual NiTi K-files	75 primary molars in children aged 4–7 years, 25 per group	Instrumentation time, obturation time, quality of obturation, postoperative pain	Rotary files (Pedo-Flex and Kedo-S) had significantly less instrumentation and obturation time and better obturation quality than manual files. Both rotary groups had less postoperative pain.	Pediatric rotary files offer superior efficiency, quality, and reduced post-operative pain vs manual files; no difference between Pedo-Flex and Kedo-S	Short-term follow-up; used 2D radiographs instead of CBCT; no microbial or long-term evaluation conducted
Kaushik et al. (2022) [91]	India	RCT	H-files, Flexicon X7, Hyflex CM	60 primary molars	Instrumentation time, obturation quality	Flexicon X7 had shortest instrumentation time	Rotary systems reduce treatment time	Used Metapex; long-term success not assessed
Lakshmanan et al. (2022) [92]	India	RCT	Manual K/H, Kedo-S Square	45 molars	Aerobic/anaerobic bacteria reduction	Kedo-S Square had 93–95% reduction	Improves microbial efficacy	No long-term clinical/radiographic outcome
Pawar et al. (2021) [93]	India/UAE/Israel	RCT	XP-endo Shaper, Kedo-S, Manual K-files	75 primary molars	Instrumentation time, obturation quality	XP-endo had best obturation and fastest time	Adaptive rotary systems show promise	Cost and complexity may limit routine use
Jeevanandan et al. (2021) [94]	India	RCT	K-files, Kedo-SH, Kedo-SG Blue	45 children	Pain, obturation (Wong-Baker scale)	SG Blue best fill (80%) and least day-1 pain	SG Blue preferred for comfort + clinical result	Pain subjective; CBCT not used for obturation
Shah et al. (2021) [95]	India	In vivo RCT	Hand K-files, Kedo-S, Pro-AF Baby GOLD	45 primary mandibular molars in 42 children aged 5–9	Instrumentation time, obturation time, radiographic quality (voids, extent)	Kedo-S: least instrumentation time (19.25 min). Pro-AF: least obturation time (4.62 min), highest optimal fill (58.5%), and lowest voids. K-files showed inferior outcomes	Pediatric rotary systems (Kedo-S, Pro-AF) significantly reduce chair-side time and improve obturation quality vs manual instrumentation	Radiographic evaluation only (2D); single operator; short-term outcomes only evaluated
Lakshmanan and Jeevanandan (2020) [96]	India	RCT	Kedo-S Square rotary file, H-file, K-file	45 primary mandibular molars (15 per group) in children aged 4–7 years	Instrumentation time (seconds), quality of obturation (Coll and Sadrian criteria)	Kedo-S Square had shortest instrumentation time (73.46 s) and highest optimal obturation rate (67%). Manual files took more time (H: 126.86 s; K: 105.6 s) and had lower obturation quality (H: 20%, K: 33%).	Kedo-S Square rotary system is superior in terms of reduced time and optimal obturation compared to traditional manual files	Short-term follow-up only; no microbial analysis; 2D radiographs limit evaluation; no long-term success data included
Ghadge et al. (2020) [97]	India	RCT	Prime Pedo, ProTaper Universal, H-files	45 primary mandibular molars	Quality of obturation, voids	Prime Pedo showed highest optimal fill and fewer voids	Pediatric rotary improves obturation outcomes	Small sample; voids radiographically assessed only
Govindaraju et al. (2017) [98]	India	RCT	K-files, ProTaper, Mtwo	45 primary mandibular molars	Time, obturation	Rotary groups faster with equal obturation	Favor rotary for time-saving	Bacterial clearance not measured
Mokhtari et al. (2017) [99]	Iran	RCT	Mtwo with apex locator vs hand	80 children	Working length accuracy, time	Rotary faster; similar WL accuracy	Efficient with apex locator	No obturation or cleaning results
Subramaniam et al. (2013) [100]	India	RCT	HERO Shaper (rotary NiTi) vs hand NiTi vs SS K-files	60 primary molars (children aged 5–9, 3 groups of 20)	Aerobic and anaerobic microbial CFU counts pre- and post-instrumentation	All 3 systems significantly reduced CFU (p<0.001); no significant difference in microbial reduction among groups	Rotary NiTi files were as effective as hand NiTi and SS files in reducing bacterial load in primary molar root canals	No long-term clinical outcomes measured; only one canal per tooth sampled (palatal/distal); lack of blinding in clinical procedures; irrigation was only saline, no antimicrobial agents used
Ochoa-Romero et al. (2011) [101]	Mexico	RCT	K3 rotary NiTi (SybronEndo) vs stainless steel hand K-files	40 primary molars (20 per group), pulpectomy in necrotic teeth	Instrumentation time, obturation time, and obturation quality (optimal, underfilled, overfilled)	Rotary significantly reduced instrumentation time (63%) and obturation time (68%). Optimal filling in 80% of rotary cases vs 50% in manual group. Statistically significant improvement (p < 0.05).	Rotary instrumentation in pulpectomy significantly improves efficiency and obturation quality in pediatric patients	Small sample size (n=20/group), single-operator bias, short follow-up duration, adult file (K3) used, not pediatric-specific system

Instrumentation Time and Efficiency

A major advantage of rotary files, consistently reported across clinical trials, was a significant reduction in instrumentation time compared to manual techniques. The data show that rotary systems reduced procedural time by approximately 26% to 78% across different studies. Saxena et al. reported that instrumentation with Pro AF Baby Gold (8.33 minutes) was 60% faster than with manual K-files (20.83 minutes) [78]. Similarly, Patel et al. found that Kedo-S Square required only 57.47 seconds, a 77.5% time reduction, compared to the 255.99 seconds needed for manual files. This enhanced efficiency contributes to shorter appointment times and may improve patient cooperation [84].

Pain and Patient Behavior

Rotary systems were associated with lower postoperative pain [77,88] and better child behavior outcomes [76,85].

Obturation Quality

Obturation outcomes assessed using radiographs or the Coll and Sadrian criteria showed higher rates of optimal fills in rotary file groups [82,84,89].

Fracture and Safety

Only one file fracture was reported in a large clinical sample using Kedo-S Plus [82], highlighting excellent durability.

Microbial Reduction and Radiographic Success

Only a few studies reported bacterial reductions [92,100], while radiographic follow-ups confirmed high success rates above 95% [87,99]. Clinical evidence across 26 studies confirms that pediatric rotary files are superior in time efficiency, comfort, and obturation quality, with favorable behavior outcomes and low complication rates.

Review Articles

A total of 19 review articles were included: 6 systematic reviews, 1 umbrella review, 1 meta-analysis, and 11 narrative reviews [6,102-119]. These reviews analyzed shaping ability, procedural time, safety, and canal adaptation (Table 5).

**Table 5:** Summary of review articles evaluating pediatric rotary endodontic systems This table compiles systematic reviews, meta-analyses, umbrella reviews, and narrative reviews that assess the design evolution, efficacy, clinical performance, and adoption of pediatric-specific rotary endodontic file systems in primary teeth. It highlights key comparisons between manual and rotary instrumentation, addresses evidence gaps, and reflects expert consensus on file selection and usage in pediatric endodontics. CBCT, cone-beam computed tomography; GPs, general practitioners; GRADE, Grading of Recommendations, Assessment, Development and Evaluations; KAP, knowledge, attitude, practices; MA, meta-analysis; NiTi, nickel-titanium; RCT, randomized controlled trial; RoB, risk of bias; SAF, self-adjusting file; SEM, scanning electron microscopy; SR, systematic review

Author (Year)	Country	Study Design	File Systems Evaluated	Sample/Model	Outcome Measures Assessed	Key Findings	Conclusion/Clinical Implication	Remarks
Patnana et al. (2025) [102]	India	Umbrella review	Hand vs rotary files	6 SRs/MAs included	Time, obturation, RoB	Rotary faster (3.2 min), but evidence low certainty	Advocates for more RCTs on pediatric rotary	GRADE scores low; RoB concerns
Sulaiman et al. (2025) [103]	India	Systematic review	ProTaper, WaveOne Gold, Reciproc, Kedo-S, SAF, OneShape, XP-Endo Shaper	5 in vitro studies on extracted primary teeth	Dentinal microcrack formation (via micro-CT, SEM, stereomicroscope)	Rotary systems with greater taper (ProTaper, XP-Endo, Reciproc) produced more cracks; SAF and Kedo-S systems showed fewer cracks; cracks most frequently occurred 3 mm from apex; hand files caused fewer cracks but are slower	Pediatric-specific rotary systems such as Kedo-S, SAF preserve dentin better than aggressive adult files	Only in vitro studies included; lacks meta-analysis; variable methodologies across studies; unclear generalizability to clinical settings
Gala et al. (2024) [6]	India	Systematic review and meta-analysis	Rotary (Kedo-S, Kedo-SG, ProTaper, XP-Endo, LightSpeed) vs hand Files (K-files, H-files)	9 RCTs (495 children, primary teeth)	Instrumentation time, quality of obturation	Rotary systems showed significantly reduced instrumentation time compared to manual files (pooled effect: 10.37 minutes; 95% CI: 8.23–12.51; p < 0.001). Pediatric-specific files such as Kedo-S also reduced postoperative pain.	Rotary files, especially pediatric-specific systems, offer superior clinical efficiency and better obturation quality in pulpectomies	Heterogeneous methodologies, limited databases searched, most studies from India, no long-term outcome meta-analysis
Tiwari et al. (2024) [104]	India	Cross-sectional + review	Pediatric rotary files (general discussion); no direct brand tested	108 dentists (54 pediatric, 54 general); literature synthesis	KAP; awareness of pediatric tools	High awareness of pediatric-specific tools but low actual use among general dentists. 87% of pedodontists and 83% of GPs acknowledged increased perforation risk with adult rotary files. Pediatric tools were preferred for improved safety, comfort, and visibility.	Kid-sized dentistry improves clinical efficiency, safety, and patient comfort; should be promoted through education and professional advocacy.	Based on survey and narrative; lacks direct clinical or in vitro evidence for rotary instrumentation performance; geographically limited to Jabalpur zone.
Parmar and Agrawal (2024) [105]	India	Narrative review	Kedo-series, Prime Pedo, Denco	Literature	Evolution, usage trends	Pediatric files improve quality and reduce time	Technological progress supports rotary adoption	Not systematic; lacks data quality assessment
Gumro et al. (2023) [106]	India	Narrative review	Kedo-S, Kedo-SG, Kedo-SG Blue, Kedo-S Square, Pro-AF Baby Gold, Prime Pedo, DXL-Pro™, Sani Kid, Fanta	Literature overview	Evolution, design features, advantages and disadvantages	Pediatric rotary files offer improved cleaning, controlled taper, reduced chair time, better adaptation in curved/narrow canals; each file system has unique metallurgy and flexibility properties	Pediatric rotary instrumentation is superior to manual; clinician must choose based on anatomy and case	Descriptive only; lacks comparative or quantitative evaluation; relies on secondary references
Kaushik et al. (2023) [107]	India	Narrative review	Kedo-S, Denco, AF Baby, etc.	Literature overview	Evolution, specs, outcomes	Pediatric files are more flexible and child-friendly	Trend favors rotary-specific pediatric files	No systematic grading; descriptive only
Haridoss et al. (2022) [108]	India	Systematic review	Reciprocating vs rotary vs hand files	12 in vitro studies	Time, canal transportation	No significant time or shaping difference between rotary types	Rotary/reciprocating better than hand	Only lab studies; no clinical RCTs included
Kaushal et al. (2022) [109]	India	Systematic review	SAF, ProTaper Next, others	7 in vitro studies	Debris extrusion	Rotary less extrusive; SAF best	Rotary safer for apical region	Only lab studies; no in vivo validation
Ranjana et al. (2021) [110]	India	Narrative review	Kedo, Pro AF Baby Gold, Pedoflex (Neoendo)	Literature synthesis	Innovations in pediatric rotary systems	Pediatric rotary systems improve cleaning, are child-specific in taper/design, and reduce procedural time	Rotary systems like Kedo and Pro AF Baby Gold are efficient and safe in primary teeth	Descriptive overview only; lacks comparative data or methodology; includes general innovations
Casaña Ruiz et al. (2022) [111]	Spain	Systematic review	ProTaper, WaveOne Gold, XP-Endo Shaper, Reciproc, Kedo-S, SAF, OneShape, BioRace, TRUShape, etc.	11 studies (RCTs, in vitro, ex vivo studies) on primary teeth instrumentation	Instrument design (length, taper, diameter), instrumentation time, dentin removal, debris extrusion, obturation quality	Rotary files such as Kedo-S and XP-Endo performed better in preserving anatomy, reducing debris extrusion, and maintaining seal; Reciproc and ProTaper removed more dentin and caused more microcracks. SAF resulted in least dentin removal but more wall damage.	Pediatric-specific files (Kedo-S, XP-Endo) recommended for safety and effectiveness in primary pulpectomies	Limited number of high-quality pediatric-specific studies; heterogeneous methods across included studies; no meta-analysis performed
Devi et al. (2021) [112]	India	Narrative review	General rotary systems	Narrative review	Morphological benefits, handling advantages	Rotary systems reduce fatigue and improve canal preservation	Supports transition to rotary endo	No statistical evaluation or grading
Padmawar et al. (2021) [113]	India	Narrative review	Kedo-S, Prime Pedo, Sani Kid, Denco	Literature overview	Evolution and clinical relevance	Pediatric files improve efficiency and acceptance	Shift toward rotary is justified	Lacks systematic methodology
Parimala et al. (2021) [114]	India	Narrative review	ProTaper, FlexMaster, HERO 642, Mtwo, K3, Kedo-S, Kedo-SG, Kedo-SG Blue, Pro AF Baby, Prime Pedo, DXL-Pro, etc.	Literature review	Design, advantages, disadvantages, clinical use	Rotary instrumentation reduces working time, improves canal shaping, and enhances obturation quality. Pediatric-specific files such as Kedo-S and Pro AF Baby are better adapted to primary canal anatomy. Clinical outcomes improve with pediatric-specific files. Post-operative pain, success rates, and reduced iatrogenic errors have been reported in cited studies.	Pediatric-specific rotary systems should be preferred over adult systems in primary teeth; proper training essential	Lacks statistical synthesis or risk of bias assessment; relies heavily on secondary citations; no direct data comparison included
Pitchiah and Shivashankarappa (2020) [115]	India	Narrative review	Adult systems (ProTaper, Hero 642, FlexMaster, WaveOne, Reciproc, Mtwo, K3, ProTaper Next) Pediatric-specific (Kedo-S, Kedo-SG, SG Blue, Square, Pro AF Baby, Prime Pedo, DXL-Pro)	Literature review	Evolution and classification of rotary systems	Kedo-S files overcome anatomical and practical limitations of adult rotary systems in primary teeth. Each generation improves on taper, flexibility, and cyclic fatigue resistance. Pediatric rotary systems reduce time, increase obturation quality.	Pediatric-specific rotary files are tailored to primary canal anatomy and offer improved clinical outcomes	Descriptive review only; lacks comparative statistical analysis; based on past literature and expert commentary
Manchanda et al. (2020) [116]	Hong Kong	Systematic review + MA	Multiple rotary and manual systems	13 RCTs	Time, obturation, post-operative pain	Rotary systems reduced pain and time	Supports rotary use in children	Moderate certainty; only English studies
Jindal et al. (2020) [117]	India	Narrative review	ProTaper, K3, FlexMaster, HERO 642, Mtwo, WaveOne, Kedo-S	Literature review	File design features, shaping technique, clinical pros/cons	Rotary instruments reduce chair time and improve child cooperation. Each system has unique taper, tip, cutting design. Kedo-S is first pediatric-specific rotary file. FlexMaster, Mtwo, HERO have various taper/pitch profiles.	Rotary systems enhance efficiency and reduce errors in pediatric pulpectomy	Review lacks quantitative comparison; based largely on expert opinion; no systematic review or grading applied
Alotaibi et al. (2020) [118]	Saudi Arabia	Narrative review	NiTi Rotary Files (Kedo-S, D1, E1), Hand files	Literature overview	Root canal anatomy, instrumentation techniques, obturation materials	NiTi rotary systems offer high flexibility, reduced working time, and better adaptation in curved primary canals. Techniques vary significantly between primary and permanent teeth. MTA, iodoform-based pastes, and calcium hydroxide are commonly used.	Rotary files improve shaping and cleaning efficiency in primary root canals; material choice must align with resorption timeline	Narrative only; lacks experimental validation or quantitative data; based heavily on secondary references and case diagrams
Ahmed (2013) [119]	Malaysia	Narrative review	ProFile, ProTaper, Hero 642, FlexMaster, K3, Mtwo, Ultrasonic, Manual K-files	Compilation of clinical and in vitro studies (2000–2012)	Root morphology, EAL accuracy, instrumentation time, cleaning efficacy, irrigant interactions	Rotary NiTi reduces instrumentation time but may risk over-instrumentation. Apex locators effective even in resorbed roots. Irrigant interactions (NaOCl + CHX) can form harmful precipitates.	Advocates rotary NiTi with caution in primary molars. Highlights need for irrigant safety protocols and careful working length determination	Review synthesizes multiple studies, but lacks quantitative meta-analysis. Not all file systems evaluated in pediatric-specific protocols. Generalizations may not reflect individual clinical variability.

Meta-analyses reported significantly shorter instrumentation times and better obturation with rotary files [6]. Umbrella and systematic reviews endorsed pediatric-specific systems such as Kedo-S, SAF, and XP-Endo, though some noted moderate to low certainty of evidence [102,103,111]. Narrative reviews emphasized evolution in file design and highlighted better acceptance of rotary instrumentation by both practitioners and children [105,115]. The review literature supports the clinical utility and efficiency of pediatric-specific rotary files, though methodological inconsistencies across included studies necessitate further high-quality research.

Case Reports and Miscellaneous Evidence

Only two case-based publications were eligible. The reports by Suresh et al. and Barr et al. described clinical success using pediatric rotary files such as Kedo-S Plus, Kedo Nano, and ProFile in primary teeth [120,121]. Although case reports are low on the evidence hierarchy, they offer real-world insights into operator file preferences and individualized canal shaping strategies (Table 6).

**Table 6:** Summary of case reports and technical notes on rotary endodontic instrumentation in primary teeth This table highlights case-based evidence and technical descriptions of rotary file systems used in pediatric endodontics. It summarizes procedural outcomes, instrumentation techniques, and shaping efficacy reported in real-world clinical settings. Although limited in generalizability, these studies demonstrate the practical use of various pediatric and adapted adult rotary files. BMP, biomechanical preparation; NiTi, nickel-titanium

Author (Year)	Country	Study Design	File Systems Evaluated	Sample/Model	Outcome Measures Assessed	Key Findings	Conclusion/Clinical Implication	Remarks
Suresh et al. (2024) [120]	India	Case report	Kedo-S Plus, Kedo Square, Kedo Nano Plus	3 primary molars	Single-visit BMP, obturation	All three files achieved optimal shaping and BMP	All generations useful depending on clinical need	Case report; not generalizable
Barr et al. (2000) [121]	USA	Technical note + case series	ProFile® NiTi rotary (.04 taper)	Case-based use in primary incisors and molars (radiographic examples only)	Descriptive: canal debridement efficiency, ease of obturation, radiographic fill	NT rotary files produced funnel-shaped preps; improved debridement and easier filling; faster and more predictable outcomes in primary teeth	Rotary instrumentation can be safely adapted for pulpectomy in primary teeth; improves fill quality and clinical efficiency	No experimental data; based on operator experience; early use of adult files in primary teeth; lacks comparative or outcome-based metrics; technique sensitive and equipment dependent

Discussion

This scoping review included 111 studies spanning over two decades (2000-2025), systematically mapping the evolution, design rationale, and clinical performance of rotary instrumentation in pediatric dentistry. The review confirms that pediatric-specific rotary systems, such as Kedo-S, Pro AF Baby Gold, Kedo-SG Blue, and Prime Pedo, have become central to clinical practice due to their efficiency, safety, and adaptation to primary tooth morphology [12-56]. The inclusion of studies from 2000-2008 adds valuable historical context, particularly highlighting early rotary file applications such as ProFile, K3, FlexMaster, and HERO 642 in primary molars [63-70]. Although these earlier systems were originally designed for permanent teeth, multiple in vitro studies demonstrated their feasibility in pediatric canals [71-73]. The comparative evaluations showed significant reductions in instrumentation time ranging from approximately 26% to 78% compared to manual hand files across the analyzed studies and acceptable shaping ability. However, these earlier systems had limitations, including a higher risk of over-preparation and less flexibility. This highlighted the clinical demand that led to the development of dedicated pediatric-specific systems, such as Kedo-S, beginning in 2016.

Evidence Synthesis: Strengths and Caveats

The evidence consistently demonstrated that rotary instrumentation, particularly systems designed for pediatric use, provided shorter instrumentation times, better shaping ability, and fewer procedural errors compared to manual files. This was reinforced by 62 in vitro studies, which showed improved canal centering (CBCT/nano-CT), less dentin removal, reduced apical debris extrusion, and more uniform obturation [12-73]. Clinical trials (n = 26) showed that rotary systems also decreased postoperative pain, enhanced child cooperation, and led to higher obturation success rates [76-101]. However, questions remain about overgeneralization of in vitro results to clinical scenarios. Many in vitro studies used resin blocks or single-rooted anterior teeth, which may not reflect the complexities of curved molar canals [16,31,33,34,51]. Moreover, a substantial number of studies relied on subjective scoring systems without microbial or longitudinal correlation. Interestingly, these earlier studies had limited evaluation of obturation quality and no assessment of microbial clearance or file fracture behavior areas that newer studies have addressed.

Alignment with Objectives

This review fulfilled its key aims by comprehensively cataloguing pediatric rotary file systems, synthesizing design features such as taper, metallurgy, and motion mechanics, and mapping clinical, biomechanical, and microbial outcomes across various study designs. However, while the review provides a thorough classification of rotary systems, some subcategories, such as reciprocating pediatric files and multi-file versus single-file systems, were underexplored due to insufficient data in the included studies.

Limitations

Several limitations were identified in this review. Over 70% of studies were from India, raising concerns about global generalizability. Differences in caries prevalence, tooth morphology, and practice models may influence outcomes elsewhere. Variable study quality was another issue, as few studies employed blinding, detailed randomization protocols, or long-term radiographic follow-up. Many in vitro models also failed to simulate clinical conditions, limiting translatability. Additionally, outcomes such as microbial reduction or fracture resistance were inconsistently reported, restricting comparisons across systems. A primary limitation, inherent to the scoping review methodology, is the absence of a formal risk of bias or GRADE assessment. While this is consistent with PRISMA-ScR guidelines, it means the certainty of evidence for specific outcomes was not formally rated. Furthermore, the included studies exhibited significant heterogeneity in their methodologies. This included varied outcome measures (e.g., different scoring criteria for obturation quality), inconsistent follow-up periods in clinical trials, and diverse in vitro models (such as extracted teeth versus resin blocks), which limits the direct comparability of findings and precluded any meta-analysis.

Strengths

This review is the first comprehensive scoping review to include 111 studies over a 25-year period and is PRISMA-ScR compliant. It integrates data across a wide evidence hierarchy from FEA simulations and imaging to clinical trials and systematic reviews. The addition of earlier studies enriched the temporal evolution and technological trajectory of pediatric file systems. Moreover, our review uniquely mapped rotary system generations, offering a visual framework to support clinical decision-making and academic discourse.

Implications for Practice and Research

The cumulative evidence justifies the clinical transition from manual to pediatric-specific rotary instrumentation. These systems offer faster, more predictable outcomes with lower procedural risk, especially in young, anxious patients. Nonetheless, from a critical standpoint, durability and cost-effectiveness of systems such as Pro AF Baby or Kedo-SG Blue remain poorly studied, and most studies excluded teeth with advanced resorption, complex canal systems, or clinical failures, which could skew success rates.

To improve evidence certainty, future research must focus on head-to-head trials comparing next-gen systems (Kedo-S Plus vs Endogal Kids vs Prime Pedo), adopt standardized outcome metrics (volumetric shaping, microbial clearance, CBCT obturation scoring), ensure longitudinal follow-up (≥12 months), include multi-ethnic populations for generalizability, and evaluate emerging adjuncts such as photodynamic therapy, AI-based working length estimation, and 3D printing for training.

## Conclusions

Rotary instrumentation has transformed pediatric endodontics, offering child-friendly systems that reduce treatment time, improve shaping outcomes, and elevate clinical predictability. This scoping review of 111 studies confirms the efficacy and safety of pediatric-specific rotary files, yet highlights the ongoing need for high-quality, globally representative trials. Clinicians are encouraged to adopt these innovations while remaining critical of study heterogeneity and mindful of individual patient anatomy. This review synthesizes over two decades of evidence, highlighting the transition from modified adult rotary files to purpose-built pediatric systems and reaffirming the clinical and biomechanical superiority of child-specific designs.
